# Piperine’s potential in treating polycystic ovarian syndrome explored through in-silico docking

**DOI:** 10.1038/s41598-024-72800-6

**Published:** 2024-09-18

**Authors:** Rahul Francis, Ramanathan Kalyanaraman, Vasuki Boominathan, Sudharsan Parthasarathy, Ashajyothi Chavaan, Irfan Aamer Ansari, Siddique Akber Ansari, Hamad M Alkahtani, Janani Chandran, Siva Vijayakumar Tharumasivam

**Affiliations:** 1 Department of Biotechnology, Srimad Andavan Arts and Science College (Autonomous), Affiliated to Bharathidasan University, Tiruchirappalli, Tamil Nadu India; 2 Department of Forestry, Nagaland University (A Central University), Lumami, Nagaland India; 3 Department of Studies in Biotechnology, Vijayanagar Sri Krishnadevarya University, Ballari, Karnataka 583-105 India; 4 Department of Drug Science and Technology, University of Turin, 10125 Turin, Italy; 5 Department of Pharmaceutical Chemistry, College of Pharmacy, King Saud University, P.O Box 2457, 11451 Riyadh, Saudi Arabia; 6 Department of Biotechnology Engineering, School of Engineering and Technology, Dhanalakshmi Srinivasan University, Samayapuram, Trichy, Tamil Nadu India

**Keywords:** PCOS, Piperine, In-silico, Docking studies, H6PD, PPARG, Cellular signalling networks, Computational models, Data processing, Virtual drug screening

## Abstract

Polycystic Ovarian Syndrome (PCOS) is a multifaceted metabolic and hormonal condition that impacts women in their procreative ages, identified by ovarian dysfunction, hyperandrogenaemia overweight and insulin insensitivity. The piperine, an important alkaloid compound of black pepper has shown promise in modulating various physiological processes. In this work, employed computational docking studies to explore the potential of piperine as a treatment for PCOS. Utilizing computational methods, we analyzed the binding interactions between piperine and key molecular targets implicated in PCOS pathogenesis, including hyperandrogenism, and “oligomenorrhea. The network pharmacology analysis report found 988 PCOS-related genes, 108 hyperandrogenism-related genes, and 377 oligomenorrhea-related genes, and we finally shortlisted 5 common genes in PCOS, hyperandrogenism, and “oligomenorrhea”: NR3C1, PPARG, FOS, CYP17A1, and H6PD. Our results reveal favorable binding affinities with PPARG (-8.34 Kcal/mol) and H6PD (-8.70 Kcal/mol) and interaction patterns, suggesting the potential of piperine to modulate these targets. Moreover, the reliability of the piperine-target interactions was revealed by molecular simulations studies. These findings support further experimental investigations to validate the therapeutic efficacy of piperine in PCOS management. The integration of computational approaches with experimental studies has the potential to lay the groundwork for the creation of new therapies specifically targeting PCOS and related endocrine disorders.

**Supplementary Information:**

The online version contains supplementary material available at 10.1038/s41598-024-72800-6.

## Introduction

Polycystic ovarian syndrome (PCOS) is a widely recognized endocrinological issue among women of conceptive age around the world^1^. The beginning of the symptomatology ordinarily happens during youth, with the side effects coming from feminine abnormalities (irregular periods), unwanted hair, obesity, insulin resistance, and related ailments^2^. In addition to a comprehensive clinical history, examination and diagnostic tests are crucial for the diagnosis of hyperandrogenism and the exclusion of alternative endocrine diseases like hyper prolactinemia or thyroid hormone abnormalities^3^. Controlling menstrual irregularities, managing hyperandrogenaemia, treating multiple comorbidities, and improving quality of life are the main goals of the pharmacological treatment of PCOS-affected patients who are unable to conceive. Overall, the pathophysiology of PCOS might be viewed as a cruel cycle of several complicated illnesses, with insulin resistance causing this complicated syndrome and hyperandrogenism acting as a predisposing factor^2^.

Nutraceutical supplements derived from plants, such as botanicals, are intricate treatments that contain a variety of phytoconstituents that can have antagonistic, agonistic, or synergistic effects within and across components^4^. Piperine is a naturally occurring compound found in Piperaceae family plants, offering potential advantages over synthetic pharmaceuticals in terms of safety, tolerability, and accessibility. Piperine is a pungent alkaloid compound present in Piperaceae family plants, which are Piper nigrum and Piper longum. In animal investigations, piperine is crucial for drug metabolism^5^. The potential therapeutic advantages of piperine might arise from its properties that include anti-inflammatory, antioxidant, and immunomodulatory effects^6^. Piperine has demonstrated potential in promoting overall metabolic health, which includes controlling inflammation, managing weight and It shows promise as a natural remedy for managing diabetes^7^. Natural compounds like piperine frequently carry a lesser risk of negative effects in comparison to synthetic pharmaceuticals^8^. By harnessing the therapeutic potential of piperine, there may be an opportunity to minimize the occurrence of side effects commonly associated with conventional PCOS treatments.

Network pharmacological analysis investigates the interactions among drugs, specific targeted proteins, medical conditions, genetic factors, and various additional factors to understand their interconnectedness and effects^9^. Using network pharmacological analysis, the modes of action of numerous drugs were evaluated. It has the potential to unveil the complex network connections among “ingredient-gene-target-disease” that aids in multi-dimensionally understanding the molecular basis of the diseases and predicting potential medication pharmacological mechanisms^4^. Nowadays, this technique is often be used for exploration of active ingredients of phyto-compounds from plants that have a wide range of medicinal uses^10^. Different networking’s can be employed to discover novel genes or elucidate the fundamental genetic mechanisms underlying polycystic ovarian syndrome (PCOS)^10^. However, the piperine network pharmacological technique hasn’t yet been used for PCOS. One important factor leading to the lack of a comprehensive and verified database for PCOS-relevant medical data, genetics and proteomics is the absence. The primary limitations include side effects, limited effectiveness in some patient subgroups, and the difficulty of maintaining lifestyle modifications. Additionally, these treatments often address only specific symptoms rather than the underlying causes of PCOS^5^. Network pharmacology offers a promising approach to overcoming the limitations of conventional PCOS treatments by providing a more comprehensive, multi-target strategy. This approach can improve the effectiveness of treatments, reduce side effects, and enable personalized medicine.

Our study, to investigate the pharmacological action of the Piperine activity in PCOS treatment. Piperine exhibits the ability to modulate multiple molecular targets implicated in PCOS pathogenesis, including hyperandrogenism, and “oligomenorrhea. This multi-targeted approach could lead to more comprehensive therapeutic effects compared to single-target interventions. Computational docking studies enable the prediction of binding interactions between piperine and specific molecular targets associated with individual PCOS phenotypes. This could aid in the creation of customized treatment plans modified to the distinctive features of each patient’s condition. The identification of piperine as a promising therapeutic agent for PCOS may stimulate further research and development in the field of nutraceuticals. Piperine-based supplements or formulations could offer convenient and non-invasive options for PCOS management. Overall, exploring piperine as a treatment for PCOS presents opportunities for the development of novel, effective, and well-tolerated pharmaceutical interventions with the potential to improve the standard of life for people who suffer from this complex endocrine disorder.

## Methodology

### Ligand preparation

Piperine’s 3D structure was supplied in the structural-data export version by the NCBI PubChem library (https://pubchem.ncbi.nlm.nih.gov/*).* The 3D structure of Piperine was energy minimized based on the Steepest descent method in SPDB Viewer software. The SwissADME web (http://www.swissadme.ch/index.php*)* server was utilised to analyse the physiochemical characteristics of piperine^11^. ADMET Characteristics of Piperine was predicted through PKCSM internet application (https://biosig.lab.uq.edu.au/pkcsm/*)*^12^.

### Identification of key drug targets in PCOS by network pharmacology approach

The DisGeNET database(https://www.disgenet.org/home/*)* was queried for genes connected with polycystic ovarian syndrome (PCOS), using “Polycystic Ovarian disease,” “Hyperandrogenomia,” and “Oligoanovalation” as search keywords. The outcomes of the three data sets were combined and de-duplicated to get genes that particularly matched the three search criteria.

### Construction of disease target genes analysis

The study employed the online Venn diagram application at (https://bioinfogp.cnb.csic.es/tools/venny/)(Oliveros, J.C.(2007–2015)to illustrate the points where disease hits meet^13^. These potential therapeutic targets were subsequently utilized to construct the KEGG Path Examination, Gene Ontology Predicting, and Protein-Protein Interacting Networks.

### Constructing interactions network of protein-protein

Using STRING (https://cn.string-db.org/*)* the intersection gene was imported and establishes the interaction connection between target proteins in order to identify the network’s primary targets^14^. Constructing a network of protein-protein interactions was built by cytoscape^15^. The degree value was determined and the core targets were selected via Cytoscape’s own analysis.

### Gene ontology’s (GO) and KEEG enrichment study

ShinyGO 0.77, an online resourcev(http://bioinformatics.sdstate.edu/go/*)*, was followed by Ge et al.., (2020) and Enrichr online tool (https://maayanlab.cloud/Enrichr/*)* followed by Kuleshov et al.., (2016) were used to examine fold enrichment analysis and significantly enhanced cellular composition, molecular action, and biological process (*P* < 0.05)^16,17^. The target’s signaling networks was examined using the KEGG database, which can be found in (https://www.genome.jp/kegg/pathway.html*).* Statistically enriched pathways (P 0.05) were found. A visual examination of the Gene ontology’s and KEGG findings was done to identify the specific PCOS treatment component^18–20^.

### Receptor preparation

Group C Nuclear Receptor Subfamily 1 Member (NR3C1)(PDB ID: 5UC1), Peroxisome Proliferators Activated Receptor Gamma (PPARG) (PDB ID: 3FUR), Transcription Factor AP-1 Subunit C-Fos (PDB ID: 1FOS), Member 1 of Cytochrome P450 Family 17 Subfamily A (CYP17A1) (PDB ID: 6WW0), and glucose 1-dehydrogenase/hexose 6-phosphate dehydrogenase (H6PD)(PDB ID: 8EM2) of Homo sapiens were selected for this study according to Network pharmacology approach .The Bank of protein Data (PDB) provided the 3Dimentional crystal forms of the receptors(www.pdb.org/pdb*)*. Version 1.13 of the UCSF-Chimera Dock preparation tool may be found at http://www.cgl.ucsf.edu/chimera utilized to clean protein receptors of heteroatoms and prepare them for docking. In the settings of the SPDB Viewer program, all receptors had their energy reduced using the steepest descent approach.

### Molecular docking study

Approach for carrying out molecular docking investigations with an adjusted flexible docking methodology by Rizvi et al. (2013)^21^. It involves using the MGL graphic tool with AutoDock Tool 4.2 Version^22^ for exploring interactions of piperine with target proteins shown in Table 1. Proteins are prepared from PDB files, and receptor grids are created, adapting to binding pockets. Docking parameters include allowing ligand rotation and selecting optimal docking postures based on RMSD, Ki, and binding energies. Cygwin 3.5.3. Version^23^ software is used for manual comparison, with ten configurations generated per protein-ligand combination. An exhaustiveness of 10 is used, and Discovery Studio 2017 is employed for post-docking analysis of ligand-protein interactions^21^.

**Table 1:** Docking Grid Box parameters used in AutoDock Software.

Protein name	PDB ID	Center grid box			Grid points		
		X	Y	Z	X	Y	Z
Member 1 of Nuclear Receptor Subfamily 3 Group C	5UC1	-24.193	9.731	31.652	60	60	60
Peroxisome Proliferator Activated Receptor Gamma	3FUR	3.965	8.302	16.054	60	60	60
Transcription Factor AP-1 Subunit C-Fos	1FOS	36.201	10.895	-14.495	60	60	60
Member 1 of Cytochrome P450 Family 17 Subfamily A	6WW0	-8.55	17.172	-42.514	60	60	60
Hexose-6-Phosphate Dehydrogenase/Glucose 1-Dehydrogenase	8EM2	159.74	157.93	160.612	60	60	60

### Molecular simulation study

The molecular simulation study applied to the docking study-identified ligand with the greatest binding ability, using AutoDock software^24^. Hits were analyzed using the Desmond 2.2 Version Program for Molecular Dynamics^25^. Each protein-ligand combination was enclosed in an orthorhombic box and hydrated using TIP3P water molecules. On addition, 0.15 M Sodium ions (Na+) and chloride ions (Cl-) were introduced into the system to neutralize it using the OPLS3e force field. The standard Desmond equilibration protocol was followed, including steps such as Brownian Dynamics NVT simulation, NPT simulation with constraints, and a final NPT simulation without constraints, each at a temperature of 10 K. The final MD simulation ran for 100 nanoseconds at standard temperature (300 K) and pressure (1.013 bars)^25^. Pressure and temperature both controlled utilizing the Nose-Hoover Chain thermometer and Martyna-Tobias-Klein barometer^26,27^. Using a smooth Particle Mesh Ewald (PME) approach with a RESPA integrator and a time step of two femtoseconds, long-distance electrostatic forces were calculated. Every 10 ps, coordinates and energy were saved for trajectory analysis.

## Results

The 3D structure of piperine was depicted in Fig. S1a. Table 2 presents the physiochemical properties of piperine, while Table S1 summarizes its ADMET properties. The ADMET properties shown that piperine has no toxicity at end points in silico models. The pkcsm tools provided predictions for a range of properties concerning piperine, including adsorption, distribution, release, waste elimination, and toxic effects represents in Table S1^28,29^.

**Table 2:** Piperine’s physicochemical characteristics.

Properties	Values
Chemical formula	C_17_H_19_NO_3_
Molecular mass	285.34 g/mol
The quantity hefty ions	21
The quantity of heavy aromatic ions	6
Csp3 portion	0.35
Quantity of movable bonds	4
Hydrogen’s bond acceptor number	3
Donors of Hydrogen’s bond	0
Atomic Refractivity	85.47
TPSA	38.77 Å^2^

### Network pharmacology protein target analysis

Polycystic ovary syndrome − 988 Genes (https://www.disgenet.org/browser/0/1/0/C0032460/*)* Hyperandrogenism − 108 Genes (https://www.disgenet.org/browser/0/1/0/C0206081/),Oligomenorrhe*a* − 37 Genes (https://www.disgenet.org/browser/0/1/0/C0028949/*).* We predicted 5 common genes in “Poly Cystic Ovary Syndrome (PCOS)”, “Hyperandrogenism” and “Oligomenorrhea”: NR3C1, PPARG, FOS, CYP17A1, H6PD. Moreover, through intersection analysis of significant pharmacological targets and genes related to PCOS, A collective of five genes has been recognized as to be viable cross-targets for PCOS treatment (Fig. S1b).

### Gene ontology’s prediction

**Fig. 1:**
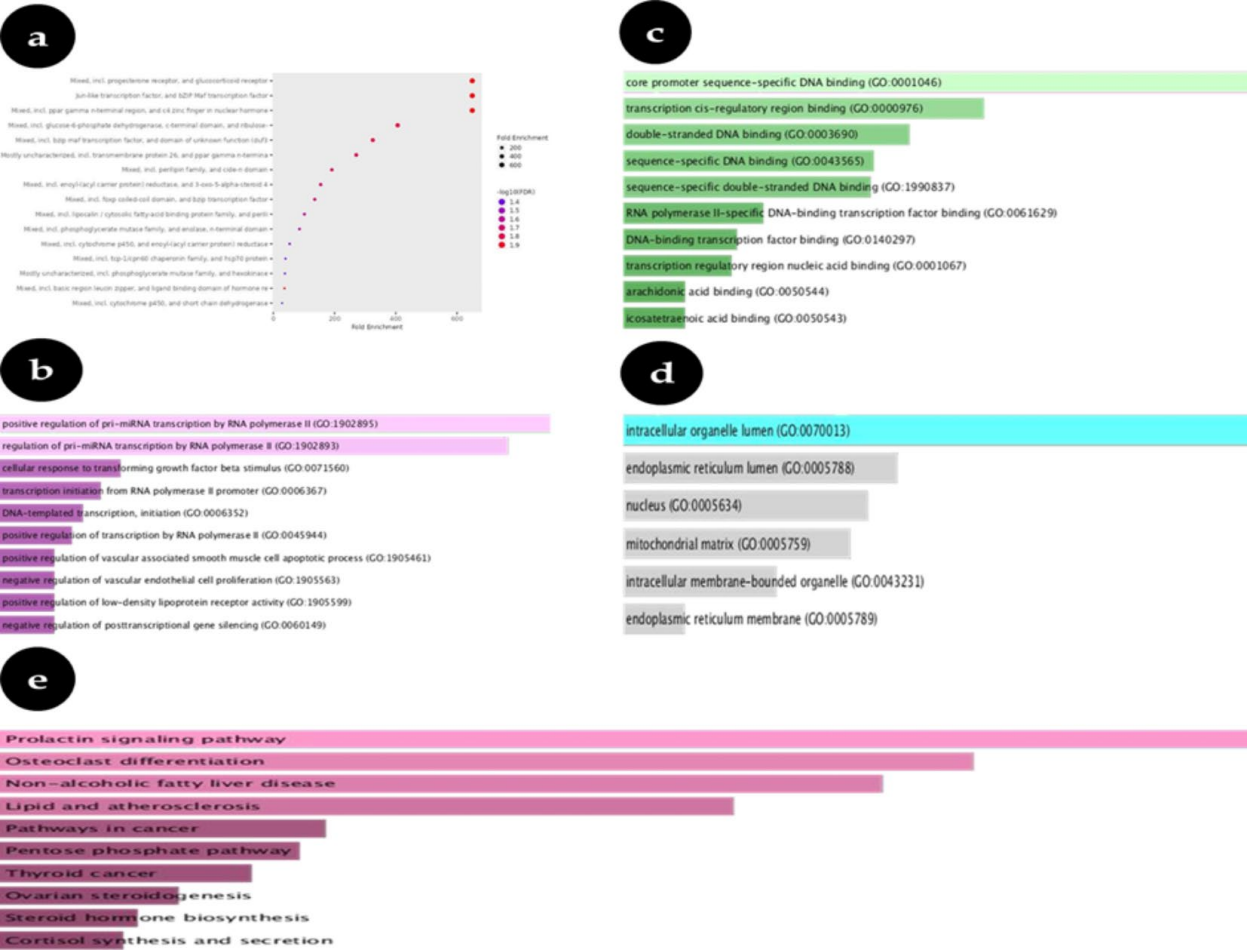
(**a**) Bubble chart of Fold enrichment chart of common protein targets in PCOS (**b**) Biological Process of common protein targets (**c**) Molecular Function Process of common protein targets (**d**) Cellular Component of common protein targets (**e**) Results of the enrichment analysis in a bar chart^18–20^.

In fold enhancement analysis, the False Detection Ratio (FDR) is calculated using the nominal P-value obtained from the hyper geometric test. In order to calculate fold enrichment, divide the proportion of your list’s genes that belong to a certain pathway by the proportion of background genes in that route. FDR reveals the probability of the enrichment occurring by chance. Large paths often have reduced FDRs because of improved statistical power. Fold Enrichment chart (Fig. 1a) shows how significantly overrepresented a certain pathway’s genes are as a metric of impact magnitude. The charts for biological activities (Fig. 1b), molecular mechanisms (Fig. 1c), and cell component (Fig. 1d) showed the projected GO keywords.

### Protein-protein interaction network analysis

We utilized PPI networks to investigate the connections that exist between various gene targets and to locate important network genes. With a confidence level of > 0.900, Homo sapiens was the chosen species, and five common targets of proteins were inserted into STRING, (Fig. S1c) shows that there were 35 nodes, 288 edges, a 16.5 typical node degree, a 0.781 the typical local clustered ratio, 87 expected edges, and a p-value of < 1.0e-16 for PPI enrichment^27^. Protein interactions involving Nuclear Receptor Subfamily 3 Group C Member 1 (NR3C1), Peroxisome Proliferator Activated Receptor Gamma (PPARG), Transcription Factor AP-1 Subunit C-Fos (FOS), Cytochrome P450 Family 17 Subfamily A Member 1 (CYP17A1), and Hexose-6-Phosphate Dehydrogenase/Glucose 1-Dehydrogenase (H6PD) were directly observed^30^. So, we predicted 5 common genes in “Poly Cystic Ovary Syndrome (PCOS)”, “Hyperandrogenism” and “Oligomenorrhea”. The five genes that are cross-targets to the piperine for the treatment of PCOS.

### KEGG enrichment pathway analysis

Several KEGG pathways were found to be closely linked with PCOS, including Prolactin signaling, Pentose phosphate pathway, Osteoclast differentiation, Thyroid cancer, endocrinal hormones biosynthesis, Non-alcohol fatty liver disease, Ovarian steroidogenesis, Lipid metabolisms and atherosclerosis, Cortisol synthesis and secretion, and Amphetamine addiction. Table S2 illustrates the 30 pathways deemed most crucial for PCOS^31^. Furthermore, Fig. 1e displays the top pathways with statistically significant differences (*P* < 0.05) as identified by the KEGG enrichment analysis.

### Molecular docking analysis

**Fig. 2:**
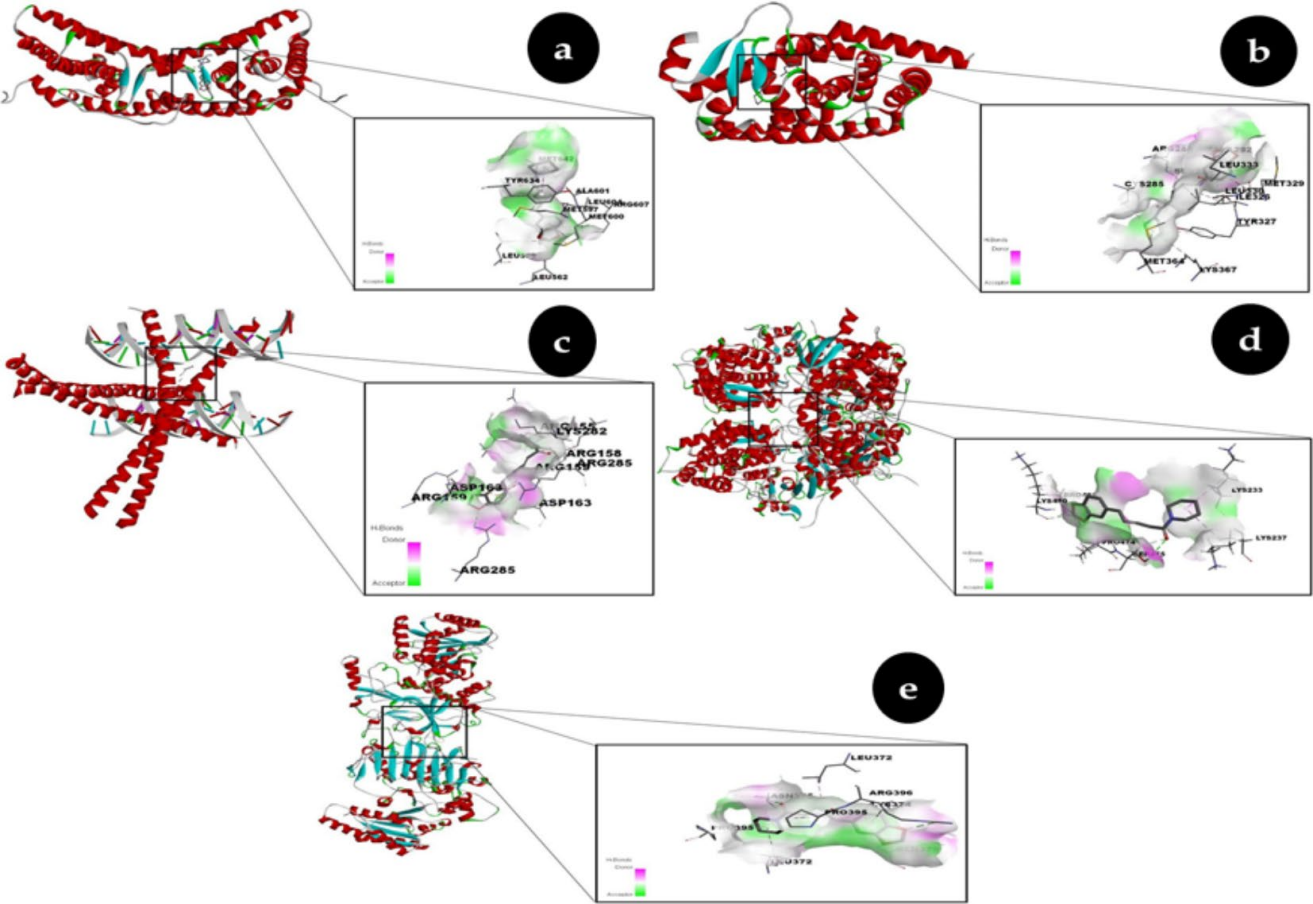
(**a**) Group C Nuclear Receptor Subfamily 1 Member in Complex with Piperine. (**b**) Piperine complex with Peroxisome Proliferator Activated Receptor Gamma (**c**) Transcription Factor AP-1 Subunit C-Fos in complex with Piperine (**d**) Cytochrome P450 Family 17 Subfamily A Member in complex with Piperine (**e**) Combined with piperine, hexose-6-phosphate dehydrogenase / glucose 1-dehydrogenase.

**Fig. 3:**
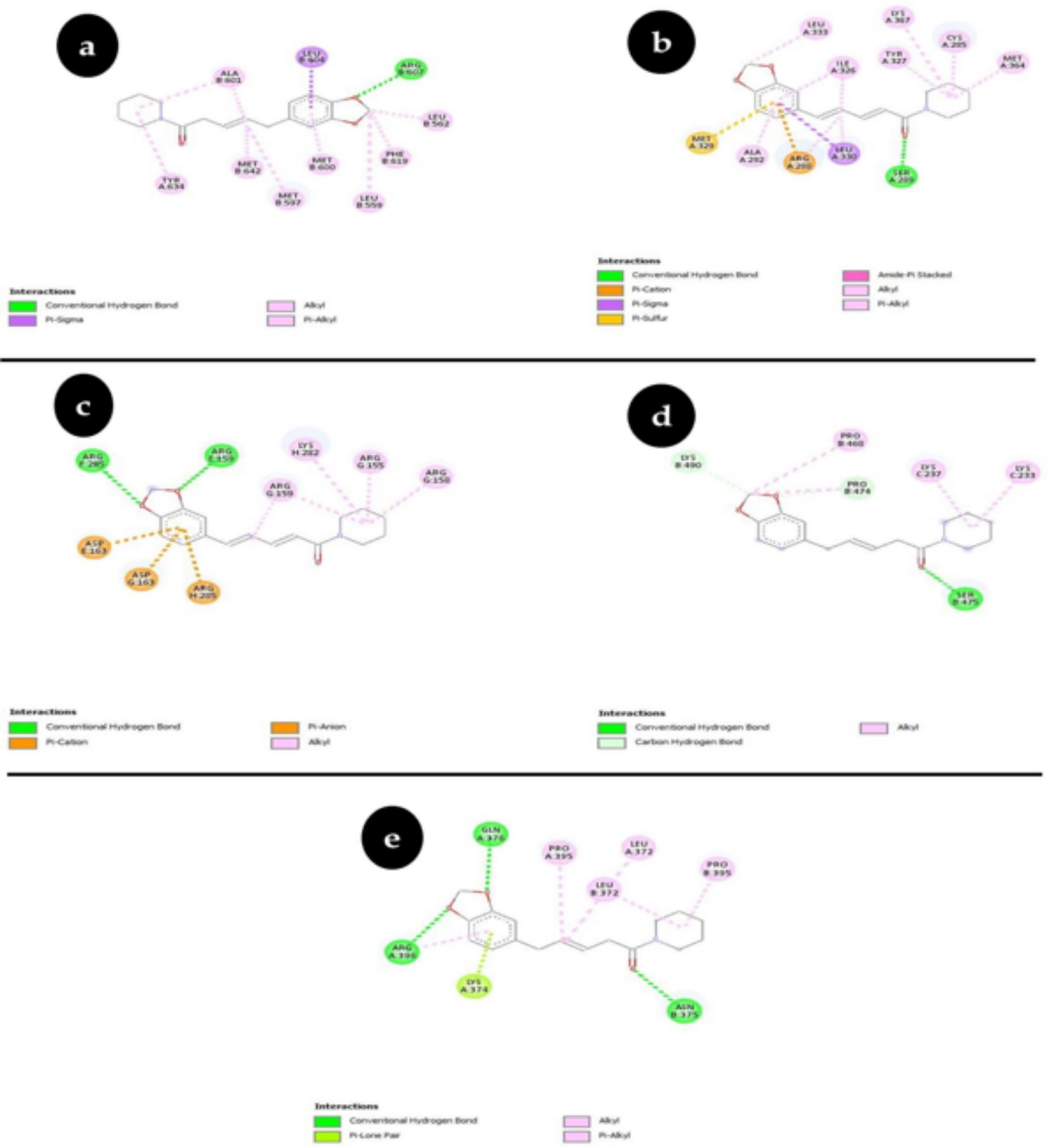
2D Interaction Plot of Docked Complexes (**a**) Group C Nuclear Receptor Subfamily 1 Member (**b**) Gamma-Activated Peroxisome Proliferator Receptor (**c**) Transcription Factor AP-1 Subunit C-Fos (**d**) Member 1 of Family 17 Subfamily A of Cytochrome P450 (**e**) Glucose 1-dehydrogenase/hexose-6-phosphate dehydrogenase (H6PD).

Following extensive validation utilizing the Lipinski rule of five targets and ADMET properties, we performed docking of piperine against the target proteins, including glucose 1-dehydrogenase/hexose-6-phosphate dehydrogenase (H6PD), Subunit C-Fos of Transcription Factor AP-1, Peroxisome Proliferators’ Activated Receptor Gamma (PPARG), Group C Nuclear Receptor Subfamily 1 Member and Member 1 of Family 17 Subfamily A of Cytochrome P450 (CYP17A1)^32^. The target and piperine’s molecular docking investigation showed that piperine had the greatest binding affinity. The piperine had − 7.96 Kcal/mol (Ki = 1.43 µM), − 8.34 Kcal/mol (Ki = 771.66 nM), -6.42 Kcal/mol (Ki = 19.77 µM), -6.43 Kcal/mol (Ki = 19.34 µM) and − 8.70 Kcal/mol (Ki = 420.17 nM) docking scores respectively show in Table 3. The docked complexes of all the protein targets were shown in (Fig. 2a–e)^32^. The 2D interaction analysis of targeted proteins with the piperine we analyzed the protein- ligand docked complexes shown in (Fig. 3a–e). This suggests that target binding necessitates the amino acid residues.

**Table 3:** Minimum binding energy scores of Piperine.

Name of the protein	PDB ID	Minimum Binding value (Kcal/mol)	Constant Inhibition (Ki)
Group C Nuclear Receptor Subfamily 1 Member	5UC1	-7.96	1.43 µM
Peroxisome Proliferator-Activated Receptor Gamma	3FUR	-8.34	771.66 nM
Transcription Factor AP-1 Subunit C-Fos	1FOS	-6.42	19.77 µM
Member 1 of Family 17 Subfamily A of Cytochrome P450	6WW0	-6.43	19.34 µM
Glucose 1-dehydrogenase/hexose-6-phosphate dehydrogenase (H6PD)	8EM2	-8.70	420.17 nM

### Molecular dynamics (MD) simulations

The Protein Data Bank (PDB) typically houses structural data of the target biomolecule obtained through X-ray or NMR techniques. Computational modeling approaches, such as homology strategies, are commonly employed to derive coordinate and geometric information regarding protein structure^33^. This step also considers the molecular environment, including solvation and ionic strength. The Maxwell-Boltzmann distributions at the optimal simulation temperature govern the initial velocities of molecules^34^. Interactions between atoms are computed using a force field, which defines both non-bonded and bonded terms based on the atomic composition, including electrostatic, Lennard-Jones, dihedral, angle, improper dihedral, and bond terms^35^.

The overall potential energy of the system is determined by accounting for all potential interactions between atoms within the protein system.

$$\begin{aligned} U\left( {\overset{\lower0.5em\hbox{$\smash{\scriptscriptstyle\rightharpoonup}$}}{R} } \right) = & \sum\limits_{{{\text{bonds}}}} {{\text{K}}_{{\text{b}}} \left( {{\text{b}} - {\text{b}}_{0} } \right)^{2} } + \sum\limits_{{{\text{angles}}}} {{\text{K}}_{{{\theta }}} \left( {{{\theta }} - {{\theta }}_{0} } \right)^{2} } + \sum\limits_{{{\text{dihedrals}}}} {{\text{K}}_{\chi } \left( {\chi - \chi _{0} } \right)} ^{2} \\ & + \sum\limits_{{{\text{nonbond}}}} {\varepsilon _{{{\text{ij}}}} \left[ {\left( {\frac{{\text{R}_{{{\text{ij}}}}^{{\min }} }}{{r_{{{\text{ij}}}} }}} \right)^{{12}} - \left( {\frac{{\text{R}_{{{\text{ij}}}}^{{\min }} }}{{r_{{{\text{ij}}}} }}} \right)^{6} } \right]} + \sum\limits_{{{\text{nonbond}}}} {\frac{{{\text{q}}_{{\text{i}}} {\text{q}}_{{\text{j}}} }}{{{\text{Dr}}_{{{\text{ij}}}} }}} \\ \end{aligned}$$

The force exerted is determined by deriving the potential energy function, equating to acceleration divided by mass according to Newton’s second law. This derivative yields a set of 3 N coupled second-order ordinary differential equations that must be solved mathematically, despite inherent complexities. The solution involves advancing positions and velocities using a numerical algorithm, iterating through time steps to generate molecular dynamics (MD) trajectories with constant energy. Coupling the system to a thermostat and employing established methods like Langevin dynamics or Nose-Hoover algorithms ensures consistent temperature dynamics^35^.

Several prominent force fields, such as CHARMM (www.charmm.org*)*, AMBER (www.ambermd.org*)*, and GROMOS (www.gromacs.org*)*, along with molecular dynamics simulation packages like NAMD (www.ks.uiuc.edu/Exploration/namd/*)* and VMD (www.ks.uiuc.edu/Exploration/vmd/*)*, significantly enhance macromolecular MD simulations^36^. Analyzing statistical properties under various initial and external conditions, such as hydrogen bond networks, hydrophobic interactions, and solvent accessible surface area, is achievable through examination of molecular dynamics trajectories^37^. By assessing the spatial relationships between hydrogen bond donors and acceptors with fixed cutoff angles and bond lengths, it becomes feasible to identify stable hydrogen bonds from transient interactions^38^. Additionally, quantifying hydrophobic stabilization effects can be achieved through solvent-accessible surface area analysis, which involves delineating surface regions using a 1.4 Å probe radius and calculating statistical metrics^39^.

Empirical energy functions, employed in creating force fields, are customizable and tailored to specific classes of molecules through parameterization. However, their applicability is limited to native systems and environments, making them unsuitable for modeling non-native systems. As a result, results often necessitate comparison with experimental data to validate accuracy and identify areas for methodological improvement. This ongoing process underscores the continuous advancement of foundational force fields and methodologies^38^.

An additional critical consideration is the ability to adequately sample the vast array of possible conformations available to even the simplest biomolecules. One potential limitation of molecular dynamics simulations is the typically restricted maximum time step usable for simulation (around 2 femtoseconds). Protein monomers’ solved structures typically encompass 40,000 atoms, while structures of dimers and membrane-bound proteins can range from 200,000 to 500,000 atoms. Achieving simulation times in the microsecond range and beyond for such large proteins with current hardware and software requires algorithmic improvements and leveraging high-performance computing resources^40^. 

### Root mean square deviation (RMSD)

The Root Mean Square Deviation (RMSD) of the protein and ligand from their reference positions in the bound complex, achieved through optimal superimposition of receptor structures, is widely accepted for assessing the accuracy of docking geometries^41^. The protein’s all-atom RMSD provides insight into how ligand binding affects the overall 3D structure. Initially, all structures were aligned based on non-hydrogen atoms, and RMSD values indicate changes in protein structure relative to a reference state. Comparisons were made with initial and average structures obtained from simulations^41^. An RMSD value ≤ 2 Å is considered optimal and recommended^42^. Interestingly, the lowest energy conformer for each ligand did not always exhibit the best pose (i.e., lowest RMSD). In some cases, a second conformer with a slightly higher binding affinity (0.0-0.5 kcal/mol) displayed a lower RMSD than the lowest energy conformer.

Initially, during a 100 ns molecular dynamics simulation, trajectories of a protein or receptor, whether in its apo form or complexed with ligands, were analyzed to assess the stability of these proteins, ligands, and their molecular interactions. A comparison was conducted between the MD trajectories of the apoprotein and the ligand-bound complex to evaluate protein fluctuations (RMSD). The graphical representation of RMSD values obtained for each ligand’s predicted lowest binding energy models were illustrated in Fig. S2.

### Protein RMSD

The plot illustrates the RMSD evolution of a protein (left Y-axis). All protein frames are initially aligned to the reference frame backbone, and RMSD is subsequently calculated based on selected atoms. The RMSD Monitoring protein provides insights into its structural conformation throughout the simulation. RMSD analysis helps determine if the simulation has equilibrated fluctuations toward the end of the simulation should reflect a thermal average structure. Changes on the order of 1–3 Å are generally acceptable for small, globular proteins. Larger changes indicate significant conformational shifts during the simulation.

Additionally, it’s crucial for the simulation to converge - RMSD values should stabilize around a fixed value. If protein RMSD continues to increase or decrease towards the end of the simulation, it suggests that equilibration hasn’t been achieved, and the simulation may require additional time for rigorous analysis.

### Ligand RMSD

The plot above displays the RMSD of a ligand (right Y-axis), indicating its stability relative to the protein and its binding pocket. In ‘Lig fit Prot’, the ligand’s RMSD is computed after aligning the protein-ligand complex to the reference protein backbone, followed by measuring the RMSD of the ligand’s heavy atoms. If the observed values are notably larger than the RMSD of the protein, it suggests that the ligand may have moved away from its initial binding site. This analysis helps assess the stability of the ligand within the protein’s environment during the simulation.

The RMSD plot for each of the four ligands is depicted in Fig. S2. In this study, RMSD values were calculated specifically for the C-alpha atoms of the protein structures. The RMSD values of the four ligands and those of the protein were compared within each complex. An acceptable and stable deviation between the RMSD of the protein alone and the RMSD of the protein-ligand complex typically ranges from one to two angstroms. RMSD serves as a measure of protein stability throughout the simulation period.

### Root mean square fluctuation (RMSF)

Following roto-translational least-squares fitting, the global structural similarity of commonly used macromolecules (proteins) is assessed by their root mean square deviation (RMSD). Conversely, experimental x-ray B-factors are utilized to focus on structural heterogeneity at the atomic level in macromolecules, providing direct information about root mean square fluctuations (RMSF) that can also be computed from molecular dynamics simulations. To evaluate the flexibility and dynamics of amino acids over a 100 ns simulation, Root Mean Square Fluctuation (RMSF) values were calculated. Each amino acid residue’s RMSF value is examined to understand its interaction dynamics with the ligand^43^.

The C-alpha backbone of the protein was analyzed based on RMSF values for each amino acid residue to further investigate the flexibility of individual residues and their interaction dynamics in ligand-bound and ligand-unbound simulations. A computational RMSF prediction indicating negative values suggests reduced fluctuations in the presence of ligands or increased fluctuations in their absence^44^. This analysis helps characterize how ligand binding influences the structural dynamics of the protein at the atomic level.

### Protein RMSF calculation

The root mean square fluctuation (RMSF) calculation provides insight into the residual mobility of amino acids. Amino acids with higher RMSF values typically indicate greater flexibility and fluctuation within the receptor structure. Conversely, those with lower RMSF values are indicative of more rigid and stable regions within the receptor.

The RMSF values of the docked complex (Hexose-6-phosphate dehydrogenase-Piperine) have been calculated and are depicted in Fig. S3a. The Fig. S3 illustrates how different amino acids within the complex exhibit varying levels of mobility, offering a detailed view of the receptor’s dynamic behavior in response to ligand binding.

When comparing to the native protein structure, the average RMSF values of receptor amino acid residues tend to be lower when bound to bioactive compounds like piperine and Hexose-6-phosphate dehydrogenase. This observation suggests that the docked ligand has contributed to the formation of a rigid and stable complex. This stability in RMSF values indicates that the ligand binding has ind uced a more constrained and structured conformation in the receptor, potentially influencing its functional behavior.

**Fig. 4:**
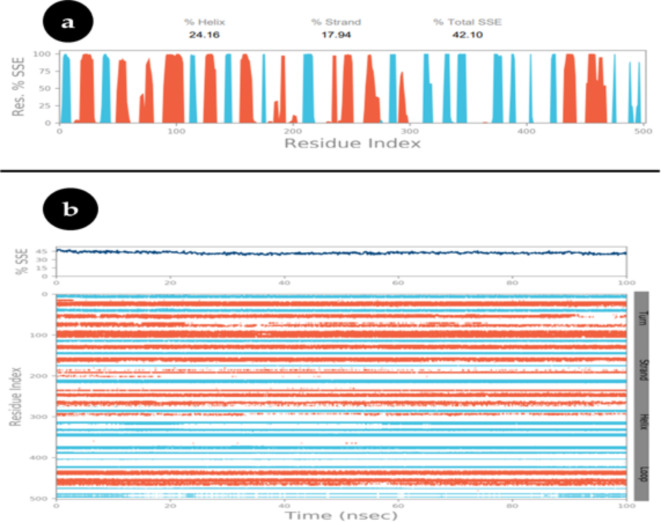
(**a**) Hexose-6-phosphate dehydrogenase (H6PD) secondary structure. The alpha helix and beta strands seen in the protein structure during the 100-ns simulation study are depicted. The plot shows there 24.16% are alpha helix, 17.94% are beta strands, and totally 42.10% are for secondary structures. (**b**) Protein secondary structure component (SSE) like as beta strands and alpha helices are constantly seen over the 100 ns simulation. Based on residue index, this graph illustrates how SSEs are distributed across structure of the protein. The SSE formation in every path structure during the MD study is summarized and at the bottom tracks the cumulative SSE assignment for every residue.

The RMSF plot of Hexose-6-phosphate dehydrogenase docked with the ligand piperine is depicted in Fig. 4. During the 100 ns MD simulation study, amino acids in the protein structure exhibited varying RMSF values, indicating fluctuation and rigidity. Specifically, residues in the ranges of 60 to 75, 250 to 300, 350 to 380, and 410 to 430 showed the highest fluctuations during the simulation. These residues are predominantly located on the cytoplasmic side of the C-terminal helix. In contrast, other regions of the protein displayed lower fluctuations, with certain areas, such as the C-terminal helix, showing less variability as depicted in the plot.

The Protein Root Mean Square Fluctuation (L-RMSF) is useful for characterizing changes in the protein and ligand atom positions. The RMSF for atom i is:

$${{RMSF}}_{{\text{i}}} {\text{ = }}\sqrt {\frac{1}{T}\sum\limits_{{t = 1}}^{T} {\left( {r_{i}^{{\prime}} (t) - r_{i} \left( {t_{{ref}} } \right)} \right)^{2} } }$$

On the RMSF plot, peaks indicate areas of the protein that exhibit the highest fluctuations during the 100 ns simulation study. Typically, the N- and C-terminal tails fluctuate more than other regions of the protein. Secondary structure elements such as alpha helices and beta strands are generally more rigid and thus exhibit lower fluctuations compared to the unstructured loop regions, which tend to be more flexible and dynamic.

#### Ligand RMSF calculation

The atom-by-atom breakdown of the ligand’s fluctuations in the Ligand RMSF plot corresponds to the 2D structure depicted in the top panel. This analysis provides insights into the entropic contributions of ligand fragments during the binding event and their interactions with the protein.

In the bottom panel, the ‘Fit Ligand on Protein’ line shows the ligand fluctuations with respect to the protein. The protein-ligand complex is first aligned based on the protein backbone, and then the ligand RMSF is measured for the ligand’s heavy atoms (Fig. S3b). This alignment allows for a detailed examination of how each part of the ligand behaves in the context of the bound complex.

The Ligand Root Mean Square Fluctuation (L-RMSF) is uses to characterize the changes present in the protein and ligand atom positions.

$${{RMSF}}_{{\text{i}}} {\text{ = }}\sqrt {\frac{1}{T}\sum\limits_{{t = 1}}^{T} {\left( {r_{i}^{{\prime}} (t) - r_{i} \left( {t_{{ref}} } \right)} \right)^{2} } }$$

where T is the trajectory time over which the RMSF is calculated, t_ref_ is the reference time, r_i_ is the position of residue i; r’ is the position of atoms in residue i after superposition on the reference, and the angle brackets indicate that the average of the square distance is taken over the selection of atoms in the residue.

### Protein-ligand contact residues

**Fig. 5:**
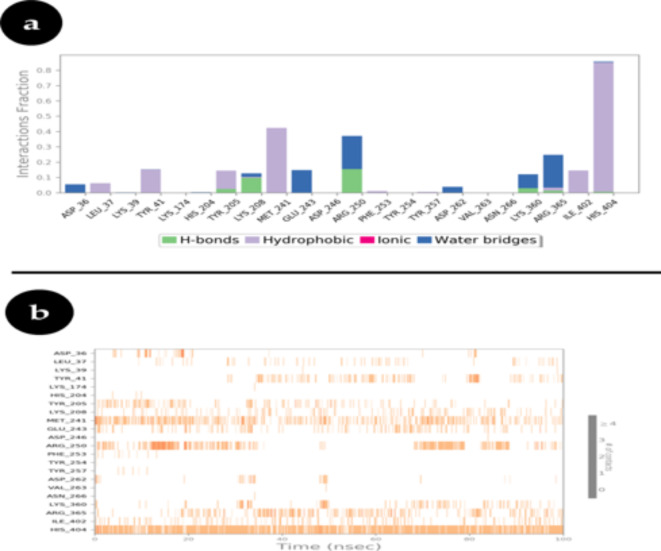
(**a**) Protein-ligand contact plots (**b**) The timeline graph displayed the interactions between the Hexose-6-phosphate dehydrogenase with Piperine in all directions in the trajectory frames (a deeper orange color indicates more than one explicit interaction with the ligand).

Protein-ligand interactions can be tracked during the simulation and are classified by type, as illustrated in Fig. 5a. Hydrogen bonds, hydrophobic contacts, ionic interactions, and water bridges are the four basic types of these interactions. The ‘Simulation Interactions Diagram’ panel can be utilized to investigate the different subtypes that are present in each category in more detail.

Throughout the simulation trajectory, the stacked bar charts in the graphic are normalized. When an interaction has a value of 0.7, for example, it means that it continues for 70% of the simulation. Since some protein residues may make several interactions of the same subtype with the ligand, values greater than 1.0 may occur.

This detailed categorization and visualization help in understanding the stability and nature of protein-ligand interactions, providing insights into the binding mechanisms and dynamics of the complex.

#### Hydrogen bond contacts

H-bonds, or hydrogen bonds, are essential for ligand binding. Because of their profound effects on drug selectivity, metabolism, and absorption, hydrogen-bonding qualities must be taken into account while designing new medications. Four subtypes of hydrogen bonds exist between a ligand and a protein: side-chain donor, side-chain acceptor, and backbone acceptor. The present geometric requirements for protein-ligand H-bonds are as follows: a donor angle of ≥ 120° between the donor-hydrogen-acceptor atoms (D—H···A); an acceptor angle of ≥ 90° between the hydrogen-acceptor-bonded atom atoms (H···A—X); and a distance of 2.5 Å between the donor and acceptor atoms (D—H···A).

Hydrogen bonding, a type of polar binding, occurs when a hydrogen atom is bound to a highly electronegative molecule and engages in electrostatic interactions with a hydrogen bond acceptor^45^. Hydrogen bonds (H-bonds) are crucial stabilizing interactions between two molecules at the binding site of a protein. To examine these interactions, the number of H-bonds is determined^46^. The H-bonds were recorded throughout the 100 ns of the receptor-ligand MD simulations (Fig. 5a).

A hydrogen bond network is a chain of hydrogen bonds connecting the side chains of multiple residues. The side chain molecules that can form hydrogen bonds are displayed in the Sidechain interaction. Molecular Dynamics (MD) simulations have also been used to explore protein hydration^47^. Hydrogen bonding is one of the most critical interactions in a biological system for stabilizing the protein-ligand complex. Figure 5b depicts the number of hydrogen bonds formed in protein-ligand complexes over the last 100 ns. The hydrogen bonding network demonstrates that these bonds are steady interactors for proteins.

In the case of the ligand (piperine) complexed with Hexose-6-phosphate dehydrogenase (H6PD), the number of intermolecular H-bonds is greater during the last 100 ns of simulation, indicating stable interaction and binding.

#### Hydrophobic contacts

Hydrophobic contacts fall into three subtypes: π-Cation; π-π; and Other, non-specific interactions. Generally these type of interactions involve a hydrophobic amino acid and an aromatic or aliphatic group on the ligand, but we have extended this category to also include π-Cation interactions. The current geometric criteria for hydrophobic interactions is as follows: π-Cation — Aromatic and charged groups within 4.5 Å; π-π — Two aromatic groups stacked face-to-face or face-to-edge; Other — A non-specific hydrophobic sidechain within 3.6 Å of a ligand’s aromatic or aliphatic carbons.

#### Ionic interaction

Ionic interactions or polar interactions, are between two oppositely charged atoms that are within 3.7 Å of each other and do not involve a hydrogen bond. We also monitor protein-metal ligand interactions, which are defined by a metal ion coordinated within 3.4 Å of protein’s and ligand’s heavy atoms (except carbon). All ionic interactions are broken down into two subtypes: those mediated by a protein backbone or side chains.

### Water bridges

Water Bridges are hydrogen-bonded protein-ligand interactions mediated by a water molecule. The hydrogen-bond geometry is slightly relaxed from the standard H-bond definition. The current geometric criteria for a protein-water or water-ligand H-bond are: a distance of 2.8 Å between the donor and acceptor atoms (D—H···A); a donor angle of ≥ 110° between the donor-hydrogen-acceptor atoms (D—H···A); and an acceptor angle of ≥ 90° between the hydrogen-acceptor-bonded atom atoms (H···A—X).

### Protein-ligand contacts timeline

A timeline representation of the interactions and contacts (H-bonds, Hydrophobic, Ionic, Water bridges) were summarized in Fig. 5a. The top panel shows the total number of specific contacts the protein makes with the ligand over the course of the trajectory. The bottom panel shows which residues interact with the ligand in each trajectory frame. Some residues make more than one specific contact with the ligand, which is represented by a darker shade of orange, according to the scale to the right of the plot (Fig. 5b).

## Ligand torsion profile

Each ligand in this study has only one rotatable bond. In our 100 ns MD simulation, the ligand (Piperine), a synthetic compound, moves without breaking its bonds. One of the movements observed is rotamerization, which involves rotations occurring around single bonds in the ligand^48^. The ligand torsion profile provides data on how well the ligand torsion aligns with theoretical estimations and indicates the preferred positions of the ligand throughout the simulation^49^. The ligand torsion profile plot is represented in Fig. S4.

This plot helps in understanding the flexibility and conformational preferences of the ligand, which are crucial for its binding affinity and specificity. By analyzing the torsion profile, we can gain insights into the stability and dynamics of the ligand within the binding site of the protein.

Plots with dials, also called radials, show the torsion conformation during the simulation. The time progression is shown radially outward from the center of the radial plot, where the simulation begins (Fig. S4).

By showing the probability density of the torsion, the bar plots give an overview of the data from the dial plot. Plotting the potential of the corresponding torsions sums up to represent the potential of the rotatable bond when torsional potential information is provided. The potential values are displayed on the chart’s left Y-axis and are given in kcal/mol. The conformational strain that the ligand undergoes in order to retain a protein-bound shape can be understood by looking at the histogram and torsion potential correlations (Fig. S4).

### Ligand (piperine) properties

I. **Ligand RMSD**: Root mean square deviation of a ligand with respect to the reference conformation (typically the first frame is used as the reference and it is regarded as time t = 0).
II. **Radius of Gyration (RoG)**: Measures the ‘extendedness’ of a ligand, and is equivalent to its principal moment of inertia. We have determined the RoG in light of the characteristic elements of apoprotein-ligand buildings (Fig. S5a). Over the 100 ns MD simulation that was investigated, all of the complexes have a constant average RoG. Such outcomes demonstrate that greater part of ligands have framed conservative and stable interaction with apoprotein when contrasted with the reference protein structure.
III. **Intramolecular Hydrogen Bonds (intraHB)**: Number of internal hydrogen bonds (HB) within a ligand molecule.
IV. **Molecular Surface Area (MolSA)**: Molecular surface calculation with 1.4 Å probe radius. This value is equivalent to a van der Waals surface area.
V. **Solvent Accessible Surface Area (SASA)**: Surface area of a molecule accessible by a water molecule. Solvent accessible surface area (SASA) of proteins has everlastingly been considered as a conclusive Fig. S5a protein unfolding and stability evaluation. The surface around a protein is portrayed by the particle’s van der Waals contact surface and a speculative focus of a dissolvable sphere^50^.
VI. **Polar Surface Area (PSA)**: Solvent accessible surface area in a molecule contributed only by oxygen and nitrogen atoms.

### MM/PB(GB)SA calculation

MM/PBSA is a famous strategy to work out calculate the binding affinities in docked complex. In the beginning, it was presented as a method with six well-defined terms and no adjustable parameters. However, since then, a lot of different options have been suggested, and the user now has to make a lot of decisions about things like the dielectric constant, the parameters for the non-polar energy, the PB or GB calculation radii, including or not including the entropy term, MD simulations, or minimizations. In practice, it typically produces results of intermediate quality, typically superior to docking and scoring but inferior to FEP; for instance, r^2^ = 0.3 for the entire PDB bind database but r^2^ = 0.0–0.8 for individual proteins. The positioning of ligands is frequently unacceptable assuming their affinities contrast by lesser than 12 kJ/mol^51^. Although it allows for greater variation in the ligand scaffold than AP methods and is less sensitive to changes in the net charge, the tested receptor–ligand system has a significant impact on the results^52^. In our study, the MM/PBSA calculation shows that the docked complex of Hexose-6-phosphate dehydrogenase/Piperine has the binding affinity of -19.71 kcal/mol (Fig. S5b).

## Discussion

PCOS is a particularly typical endocrinological illness, identified by persistent anovulation and hyperandrogenism^53^. If PCOS is not efficiently and adequately treated, it can drastically decrease life expectancy^54^. Many biomarkers have been linked to PCOS and could serve as potential treatment targets. However, the exact mechanism of gene regulation leading to the progression of the syndrome remains unclear^55^. The docking process assesses the binding affinity between molecules. In this research endeavor, we sought to investigate the therapeutic potential of piperine in treating PCOS by employing bioinformatics analysis to gain insights into their biological roles. Piperine, a natural polyphenol derived from black pepper (*Piper nigrum*) and long peppers (*Piper longum*), It’s been used as a food spice and a potential therapy for several illnesses, including inflammation, obesity, and various malignancies^56^. Piperine is an orange needle-shaped crystal with melting and lighting points of 131–132 °C. Chloroform and methanol soluble; unable to dissolve in water^57^.

Priyanka et al. (2020) found that the results of the ADMET study on piperine derivatives derived from natural sources indicated that most ADME (Absorption, Distribution, Metabolism, and Excretion) features exhibited favorable characteristics, suggesting that these compounds are promising candidates for further development^58^. In the study investigation, the NCBI PubChem database makes the 3D structure of piperine accessible in the format of a structure-data file (SDF). The 3D structure of piperine was seen in Fig. S1a. Using the SwissADME web server, the physiochemical characteristics of piperine were examined, and ADMET characteristics were predicted using the online PKCSM program. Table 2 displayed the physiochemical characteristics of piperine, while Table S1 provided a summary of its ADMET characteristics. In this studiy, we confirmed as same results through in silico ADMET models that piperine exhibits no harmful effects at end points.

Kamboj et al. utilized molecular docking to assess the binding affinity between compounds and CYP17. Their findings indicate that all phytocompounds exhibited blocking or hindrance of the CYP17 enzyme’s activity, with docked scores ranging from − 3.7 to -9.5 ^59^. Using computational molecular docking studies, Amudha studied the effectiveness of phytochemicals derived from *Cadaba fruticosa* (L.) Druce in inhibiting CYP17. Docking scores ranged from − 3.3 to -7.9, indicating that all 20 drugs demonstrated moderate to strong inhibition. In particular, docking scores of -7.9 and − 6.8 indicated strong inhibition of androstan-3-one and 17-hydroxy-2, 4-dimethyl respectively^60^. A more extensive negative value in the docking score denotes a better affinity among the ligand and the protien. The docking of ligands and proteins was ranked according to their binding affinity, with lower (more negative) scores indicating stronger interactions. Upon conducting a computational docking study, we found that piperine has the greatest affinity for binding to the targeted proteins. The docked complexes of all the protein targets are illustrated in Fig. S2 through 13. Over all, the docking study revealed that piperine exhibited the highest binding affinity with Peroxisome Proliferator Activated Receptor Gamma (-8.34 kcal/mol)^31^ and Hexose-6-Phosphate Dehydrogenase/Glucose 1-Dehydrogenase (-8.70 kcal/mol).

The expression of PPAR-γ in the ovaries has been linked to the formation of follicles and ovarian function. Changes in PPAR-γ signalling might interfere with ovarian steroidogenesis and folliculogenesis, which could lead to reproductive problems in PCOS, including irregular menstruation and infertility (Komar, 2005)^61^. In addition, Ahmadian et al. (2013) highlighted the significant function of the PPAR-γ protein in regulating adipocyte development, glucose levels, and lipid homeostasis^62^. An important nuclear receptor recognized as peroxisome proliferator-activated receptor-γ (PPAR-γ) is connected with hyperandrogenemia and plays a role to regulating energy balance, as noted by Stump et al. (2015)^63^. Chen, et al.., (2015) noted that Androgen metabolism and androgen receptor signalling have been associated with PPAR-γ. The hyperandrogenic phenotype associated with PCOS may be attributed to dysregulated PPAR-γ activation^64^. Unluturk et al. (2007) found that PPAR-γ genes are associated with PCOS occurrences across different ethnic populations. Their study provided evidence linking PPAR-γ with the pathogenesis of PCOS^65^. According to our docking study, piperine exhibits the highest binding affinity with the Gamma-Activated Peroxisome Proliferator Receptor, with a calculated energy of -8.34 kcal/mol. Overall, PPAR-γ docking studies in PCOS research give important insights into the underlying molecular processes of the illness and this could potentially lead to the development of novel treatment approaches.

Docking studies involving Hexose-6-phosphate dehydrogenase (H6PD) genes and their relevance to PCOS are not commonly reported in the literature. While H6PD is involved in various metabolic pathways and play the important role in PCOS pathophysiology, specific docking studies focusing on H6PD genes in the context of PCOS are limited^66^. However, computational docking studies involving other genes or proteins implicated in PCOS, such as PPAR-γ or insulin receptors, have been conducted to explore potential therapeutic interventions. Continuing research exploring the interaction between H6PD genes and pertinent molecular targets in PCOS could yield valuable insights into the fundamental mechanisms of the disorder.

Hexose-6-phosphate dehydrogenase (H6PD) genes play the important role in polycystic ovarian syndrome (PCOS) by influencing various metabolic pathways and hormonal regulation. These genes contribute to glucose metabolism, oxidative stress management, steroid hormone production, insulin sensitivity, and lipid metabolism, all of which are dysregulated in PCOS (Li Yan et al., 2019)^67^. Understanding the involvement of H6PD in PCOS can provide insights into the disorder’s mechanisms and potential therapeutic strategies. Cortisone reductase deficiency, caused by inactivating mutations in the enzyme Glucose 1-dehydrogenase/hexose-6-phosphate dehydrogenase (H6PD), mimics of PCOS and manifests as hyperandrogenism that cannot be explained by standard tests (Qin and Rosenfield 2011)^68^. Based on docking study, it was found that piperine demonstrates the greatest binding affinity with the Glucose 1-dehydrogenase/hexose-6-phosphate dehydrogenase (H6PD), showing a calculated energy of -8.70 kcal/mol.

Molecule Dynamic Simulation is regularly utilized to refine proteins or any molecular structures and find previously elusive information about the time evolution of conformations (Vanommeslaeghe et al., 2014)^35^. The protein-ligand RMSD showed that hexose-6-phosphate dehydrogenase docking complexes with 3.6 were present. The RMSD value of piperine when it binds to hexose-6-phosphate dehydrogenase (3.6) is larger than in the natural crystal structure of Glucose 1-dehydrogenase/hexose-6-phosphate dehydrogenase (H6PD). To confirm the adaptability and flexibility of amino acids during simulation for 100 nano seconds, the root of mean squares change (RMSF) was determined. Every residue of the amino acid RMSF value is checked as it cooperates with the ligand along a direction (De Vita et al., 2021)^69^. A well-known method for calculating the binding affinities of docked complexes is MM/PBSA. According to our study’s MM/PBSA calculations, the docked complex of hexose-6-phosphate dehydrogenase and piperine exhibits a binding affinity of -19.71 kcal/mol, as seen in Fig. S3b.

## Conclusion

In conclusion, the combination of network pharmacology and molecular docking analyses offers empirical evidence supporting the potential pharmaceutical application of exploring the potential role of piperine in the treatment or management of polycystic ovarian syndrome (PCOS). Through computational analyses, piperine exhibited promising drug-like characteristics and demonstrated favorable interactions with key proteins associated with PCOS pathogenesis, particularly Hexose-6-phosphate dehydrogenase. Molecular dynamics simulations further confirmed the stability and efficacy of the piperine-Hexose-6-phosphate dehydrogenase complex, suggesting its potential as a therapeutic agent for managing PCOS symptoms. These findings lay a solid scientific foundation for further exploration and development of piperine-based treatments for PCOS.

## Electronic supplementary material

Below is the link to the electronic supplementary material.

Supplementary Material 1

## Data Availability

The datasets used and/or analysed during the current study available from the corresponding author on reasonable request.

## References

[1] Bozdag, G. *et al.* The prevalence and phenotypic features of polycystic ovary syndrome: A systematic review and meta-analysis. *Hum. Reprod. Open* **31**(12), 2841–2855 (2016). 10.1093/humrep/dew218 27664216

[2] Azziz, R. *et al.* Polycystic ovary syndrome. *Nat. Rev. Dis. Primers.* **2**, 16057. 10.1038/nrdp.2016.57 (2016). 27510637 10.1038/nrdp.2016.57

[3] Al Wattar, B. H. *et al.* Clinical practice guidelines on the diagnosis and management of polycystic ovary syndrome: A systematic review and quality assessment study. *J. Clin. Endocrinol. Metab.* **106**(8), 2436–2446 (2021). 33839790 10.1210/clinem/dgab232 PMC8830055

[4] Tiwari, A. *et al.* Network pharmacology-based strategic prediction and target identification of apocarotenoids and carotenoids from standardized Kashmir saffron (*Crocus**sativus* L.) extract against polycystic ovary syndrome. *Medicine* **102**(32), e34514 (2023). 37565925 10.1097/MD.0000000000034514 PMC10419424

[5] Daniel, N. *et al.* Phytochemical, cytotoxicity and antioxidant activities of the stem bark of Piper arborescens. *MJFAS* **13**(4), 840–845 (2017).

[6] Yadav, S. S. *et al.* Therapeutic spectrum of piperine for clinical practice: A scoping review. *Crit. Rev. Food Sci. Nutr.* **63**(22), 5813–5840 (2023). 34996326 10.1080/10408398.2021.2024792

[7] Prasad, M. *et al.* Piperine modulates IR/Akt/GLUT4 pathways to mitigate insulin resistance: Evidence from animal and computational studies. *Int. J. Biol. Macromol.* **253**, 127242 (2023). 37797864 10.1016/j.ijbiomac.2023.127242

[8] Muddapur, U. M. *et al.* Exploring bioactive phytochemicals in Gymnema sylvestre: Biomedical uses and computational investigations. *Separations* **11**(2), 50 (2024).

[9] Hopkins, A. L. Network pharmacology: The next paradigm in drug discovery. *Nat. Chem. Biol.* **4**(11), 682–690 (2008). 18936753 10.1038/nchembio.118

[10] Hossain, M. A. *et al.* Molecular docking and pharmacology study to explore bio-active compounds and underlying mechanisms of Caesalpinia bonducella on polycystic ovarian syndrome. *IMU* **33**, 101073 (2022).

[11] Daina, A., Michielin, O. & Zoete, V. Swiss ADME: A free web tool to evaluate pharmacokinetics, drug-likeness and medicinal chemistry friendliness of small molecules. *Sci. Rep.* **7**(1), 42717 (2017). 28256516 10.1038/srep42717 PMC5335600

[12] Pires, D. E., Blundell, T. L. & Ascher, D. B. Pk CSM: Predicting small-molecule pharmacokinetic and toxicity properties using graph-based signatures. *J. Med. Chem.* **58**(9), 4066–4072 (2015). 25860834 10.1021/acs.jmedchem.5b00104 PMC4434528

[13] Venny, O. J. An interactive tool for comparing lists with Venn Diagrams. http://bioinfogp.cnb.csic.Es/tools/venny/index.Html. (2007).

[14] Feng, S. H. *et al.* The mechanism of Bushen Huoxue decoction in treating intervertebral disc degeneration based on network pharmacology. *Ann. Palliat. Med.* **10**(4), 3783792 (2021). 10.21037/apm-20-2586 33752429

[15] Zhang, B. *et al.* Human placental cytotrophoblast epigenome dynamics over gestation and alterations in placental disease. *Dev. Cell.* **56**(9), 1238–1252 (2021). 33891899 10.1016/j.devcel.2021.04.001 PMC8650129

[16] Ge, S. X. *et al.* A graphical gene-set enrichment tool for animals and plants. *Bioinformatics* **36**(8), 2628–2629 (2020). 31882993 10.1093/bioinformatics/btz931 PMC7178415

[17] Kuleshov, M. V. *et al.* A comprehensive gene set enrichment analysis web server 2016 update. *Nucl. Acids Res.* **44**(1), W90–W97 (2016). 27141961 10.1093/nar/gkw377 PMC4987924

[18] Kanehisa, M. & Goto, S. KEGG: Kyoto encyclopedia of genes and genomes. *Nucl. Acids Res.* **28**(1), 27–30 (2000). 10592173 10.1093/nar/28.1.27 PMC102409

[19] Kanehisa, M. Toward understanding the origin and evolution of cellular organisms. *Prot. Sci.* **28**(11), 1947–1951 (2019). 10.1002/pro.3715 PMC6798127 31441146

[20] Kanehisa, M., Furumichi, M., Sato, Y., Kawashima, M. & Ishiguro-Watanabe, M. KEGG for taxonomy-based analysis of pathways and genomes. *Nucl. Acids Res.* **51**(D1), D587–D592 (2023). 36300620 10.1093/nar/gkac963 PMC9825424

[21] Rizvi, S. M., Shakil, S. & Haneef, M. A simple click by click protocol to perform docking: AutoDock 4.2 made easy for non-bioinformaticians. *EXCLI J.* **12**, 831 (2013). 26648810 PMC4669947

[22] Huey, R., Morris, G. M. & Forli, S. Using AutoDock 4 and AutoDock vina with AutoDockTools: A tutorial. *Scripps Res. Instit. Mol. Graph. Lab.* **10550**(92037), 1000 (2012).

[23] Lazenby, D. Cygwin: For windows NT. *Linux J* **75**, 14-es (2000).

[24] Mol, G. S. *et al.* Modeling the structural and reactivity properties of capsaicin [(E)-N-[(4-hydroxy-3-methoxyphenyl) methyl]-8-methylnon-6-enamide] wavefunction-dependent properties, pharmacokinetics, in-silico analysis, and molecular dynamics simulation. *J. Mol. Struct.* **1304**, 137591 (2024).

[25] Bowers, K. J., Chow, E., Xu, H., Dror, R. O., Eastwood, M. P., Gregersen, B. A., Klepeis, J.L., Kolossvary, I., Moraes, M. A., Sacerdoti, F. D. & Salmon, J. K. Scalable algorithms for molecular dynamics simulations on commodity clusters. in *Proceedings of the 2006 ACM/IEEE Conference on Supercomputing*, p. 84 (2006)

[26] Rehman, M. T., AlAjmi, M. F. & Hussain, A. Natural compounds as inhibitors of SARS-CoV-2 main protease (3CLpro): A molecular docking and simulation approach to combat COVID-19. *Curr. Pharm. Des.* **27**(33), 3577–3589 (2021). 33200697 10.2174/1381612826999201116195851

[27] Martyna, G. J., Tobias, D. J. & Klein, M. L. Constant pressure molecular dynamics algorithms. *J. Chem. Phys.* **101**(5), 4177–4789 (1994).

[28] Raza, H. *et al.* Isolation, characterization, and in silico, in vitro and in vivo antiulcer studies of isoimperatorin crystallized from Ostericum koreanum. *Pharm. Biol.* **55**(1), 218–226 (2017). 27927061 10.1080/13880209.2016.1257641 PMC6130598

[29] Poleboyina, P. K. *et al.* Virtual screening, molecular docking, and dynamic simulations revealed TGF-β1 potential inhibitors to curtail cervical cancer progression. *Appl. Biochem. Biotechnol.* **196**(3), 1316–1349 (2024). 37392324 10.1007/s12010-023-04608-5

[30] Singh, A. K. *et al.* A network pharmacology approach with experimental validation to discover protective mechanism of poly herbal extract on diabetes mellitus. *J. King Saud Univ. Sci.* **20**, 103138 (2024).

[31] Kanehisa, M. *Post-genome informatics* 20 (OUP Oxford, 2000).

[32] Rankinen, T. *et al.* The human obesity gene map: The 2005 update. *Obesity.* **14**(4), 529–644 (2006). 16741264 10.1038/oby.2006.71

[33] Haddad, Y., Adam, V. & Heger, Z. Ten quick tips for homology modeling of high-resolution protein 3D structures. *PLoS Comput. Biol.* **16**(4), e1007449 (2020). 32240155 10.1371/journal.pcbi.1007449 PMC7117658

[34] Braun, E. et al. Best practices for foundations in molecular simulations [Article v1.0]. *Living J. Computat. Mol. Sci.* **1**(1), 5957. 10.33011/livecoms.1.1.5957 (2019). 10.33011/livecoms.1.1.5957 PMC6884151 31788666

[35] Vanommeslaeghe, K., Guvench, O. & MacKerell, A. D. Jr. Molecular mechanics. *Curr. Pharmaceut. Design* **20**(20), 3281–3292. 10.2174/13816128113199990600 (2014). 10.2174/13816128113199990600 PMC4026342 23947650

[36] Lorenz, C. & Doltsinis, N. Molecular dynamics simulation: From “Ab Initio” to “coarse grained.” In *Handbook of computational chemistry* (ed. Leszczynski, J.) (Springer, 2015). 10.1007/978-94-007-6169-8_7-2.

[37] Shih, A. J., Telesco, S. E., Choi, S. H., Lemmon, M. A. & Radhakrishnan, R. Molecular dynamics analysis of conserved hydrophobic and hydrophilic bond-interaction networks in ErbB family kinases. *Biochem. J.* **436**(2), 241–251. 10.1042/BJ20101791 (2011). 21426301 10.1042/BJ20101791 PMC3138537

[38] Chen, D. et al. Regulation of protein-ligand binding affinity by hydrogen bond pairing. *Sci. Adv.* **2**(3), e1501240. 10.1126/sciadv.1501240 (2016). 27051863 10.1126/sciadv.1501240 PMC4820369

[39] Durham, E., Dorr, B., Woetzel, N., Staritzbichler, R. & Meiler, J. Solvent accessible surface area approximations for rapid and accurate protein structure prediction. *J. Mol. Model.* **15**(9), 1093–1108. 10.1007/s00894-009-0454-9 (2009). 19234730 10.1007/s00894-009-0454-9 PMC2712621

[40] Hollingsworth, S. A. & Dror, R. O. Molecular dynamics simulation for all. *Neuron* **99**(6), 1129–1143. 10.1016/j.neuron.2018.08.011 (2018). 30236283 10.1016/j.neuron.2018.08.011 PMC6209097

[41] Kufareva, I. & Abagyan, R. Methods of protein structure comparison. In *Methods in molecular biology* Vol. 857 231–257 (Human press, 2012). 10.1007/978-1-61779-588-6_10. 10.1007/978-1-61779-588-6_10 PMC4321859 22323224

[42] Castro-Alvarez, A., Costa, A. M. & Vilarrasa, J. The performance of several docking programs at reproducing protein-macrolide-like crystal structures. *Molecules (Basel, Switzerland)* **22**(1), 136. 10.3390/molecules22010136 (2017). 28106755 10.3390/molecules22010136 PMC6155922

[43] De Vita, S., Chini, M. G., Bifulco, G. & Lauro, G. Insights into the ligand binding to bromodomain-containing protein 9 (BRD9): A guide to the selection of potential binders by computational methods. *Molecules (Basel, Switzerland)* **26**(23), 7192. 10.3390/molecules26237192 (2021). 34885774 10.3390/molecules26237192 PMC8659208

[44] Milano, T., Gulzar, A., Narzi, D., Guidoni, L. & Pascarella, S. Molecular dynamics simulation unveils the conformational flexibility of the interdomain linker in the bacterial transcriptional regulator GabR from Bacillus subtilis bound to pyridoxal 5’-phosphate. *PloS one* **12**(12), e0189270. 10.1371/journal.pone.0189270 (2017). 29253008 10.1371/journal.pone.0189270 PMC5734734

[45] Simončič, M. & Urbič, T. Hydrogen bonding between hydrides of the upper-right part of the periodic table. *Chem. Phys.* **507**, 34–43. 10.1016/j.chemphys.2018.03.036 (2018). 30364625 10.1016/j.chemphys.2018.03.036 PMC6197068

[46] Pace, C. N. et al. Contribution of hydrogen bonds to protein stability. *Prot. Sci.: A Publ. Prot. Soc.* **23**(5), 652–661. 10.1002/pro.2449 (2014). 10.1002/pro.2449 PMC4005716 24591301

[47] Petukhov, M., Rychkov, G., Firsov, L. & Serrano, L. H-bonding in protein hydration revisited. *Prot. Sci.: A Publ. Prot. Soci.* **13**(8), 2120–2129. 10.1110/ps.04748404 (2004). 10.1110/ps.04748404 PMC2279814 15238635

[48] McNaught, A. D. & Wilkinson, A. Compendium of chemical terminology: IUPAC recommendations. (No Title) (1997).

[49] Hao, M. H., Haq, O. & Muegge, I. Torsion angle preference and energetics of small-molecule ligands bound to proteins. *J. Chem. Inform. Model.* **47**(6), 2242–2252 (2007). 10.1021/ci700189s 17880058

[50] Ali, S. A., Hassan, M. I., Islam, A. & Ahmad, F. A review of methods available to estimate solvent-accessible surface areas of soluble proteins in the folded and unfolded states. *Curr. Prot. Pept. Sci.* **15**(5), 456–476. 10.2174/1389203715666140327114232 (2014). 10.2174/1389203715666140327114232 24678666

[51] Sun, H., Li, Y., Tian, S., Xu, L. & Hou, T. Assessing the performance of MM/PBSA and MM/GBSA methods. 4. Accuracies of MM/PBSA and MM/GBSA methodologies evaluated by various simulation protocols using a PDBbind data set. *Phys. Chem. Chem. Phys.* **16**(31), 16719–16729 (2014). 24999761 10.1039/c4cp01388c

[52] Genheden, S. & Ryde, U. The MM/PBSA and MM/GBSA methods to estimate ligand-binding affinities. *Expert Opinion Drug Discov.* **10**(5), 449–461. 10.1517/17460441.2015.1032936 (2015). 10.1517/17460441.2015.1032936 PMC4487606 25835573

[53] Paczkowska, K. *et al.* Alteration of branched-chain and aromatic amino acid profile as a novel approach in studying polycystic ovary syndrome pathogenesis. *Nutrients* **15**(19), 4153 (2023). 37836437 10.3390/nu15194153 PMC10574162

[54] Devarbhavi, P. *et al.* Identification of key pathways and genes in polycystic ovary syndrome via integrated bioinformatics analysis and prediction of small therapeutic molecules. *Reprod. Biol. Endocrinol.* **19**(1), 31 (2021). 33622336 10.1186/s12958-021-00706-3 PMC7901211

[55] Carvalho, L. M. *et al.* Microparticles: Inflammatory and haemostatic biomarkers in polycystic ovary syndrome. *Mol. Cell. Endocrinol.* **5**(443), 155–162 (2017). 10.1016/j.mce.2017.01.017 28088464

[56] Ren, T. *et al.* Piperine-loaded nanoparticles with enhanced dissolution and oral bioavailability for epilepsy control. *Eur. J. Pharm. Sci.* **137**, 104988 (2019). 31291598 10.1016/j.ejps.2019.104988

[57] Paarakh, P. M., Sreeram, D. C. & Ganapathy, S. P. In vitro cytotoxic and in silico activity of piperine isolated from Piper nigrum fruits Linn. *In Silico Pharmacol.* **3**, 1–7 (2015). 26820894 10.1186/s40203-015-0013-2 PMC4731375

[58] Dhiman, P., Malik, N. & Khatkar, A. Natural based piperine derivatives as potent monoamine oxidase inhibitors: An in silico ADMET analysis and molecular docking studies. *BMC chem.* **14**, 1–6 (2020). 32099971 10.1186/s13065-020-0661-0 PMC7027018

[59] Kamboj A, *et al.* A Molecular Docking Study towards Finding Herbal Treatment against Polycystic Ovary Syndrome (PCOS).

[60] Amudha, M. & Rani, S. In silico molecular docking studies on the phytoconstituents of cadaba fruticosa (L.) druce for its fertility activity. *Asian J. Pharm. Clin. Res.* **1**, 48–50 (2016).

[61] Komar, C. M. Peroxisome proliferator-activated receptors (PPARs) and ovarian function–implications for regulating steroidogenesis, differentiation, and tissue remodeling. *Reprod. Biol. Endocrinol.* **3**, 1–4 (2005). 16131403 10.1186/1477-7827-3-41 PMC1266036

[62] Ahmadian, M. *et al.* PPARγ signaling and metabolism: The good, the bad and the future. *Nat. Med.* **19**(5), 557–566 (2013). 23652116 10.1038/nm.3159 PMC3870016

[63] Stump, M. *et al.* PPARγ regulation in hypertension and metabolic syndrome. *Curr. Hypertens. Rep.* **17**(12), 89 (2015). 26462805 10.1007/s11906-015-0601-x PMC6766749

[64] Chen, M. J. *et al.* The effect of androgens on ovarian follicle maturation: Dihydrotestosterone suppress FSH-stimulated granulosa cell proliferation by upregulating PPARγ-dependent PTEN expression. *Sci. Rep.* **5**(1), 18319 (2015). 26674985 10.1038/srep18319 PMC4682139

[65] Unluturk, U. *et al.* The genetic basis of the polycystic ovary syndrome: a literature review including discussion of PPAR-γ. *PPAR res*. 10.1155/2007/49109 (2007). 10.1155/2007/49109 PMC1820621 17389770

[66] Naseri, R. *et al.* H6PD gene polymorphisms (R453Q and D151A) and polycystic ovary syndrome: A case-control study in a population of Iranian Kurdish Women. *Int. j. fertil. steril.* **16**(3), 180 (2022). 36029054 10.22074/IJFS.2021.141690.1050 PMC9396008

[67] Li, Y. *et al.* Multi-system reproductive metabolic disorder: significance for the pathogenesis and therapy of polycystic ovary syndrome (PCOS). *Life Sci.* **1**(228), 167–175 (2019). 10.1016/j.lfs.2019.04.046 31029778

[68] Qin, K. & Rosenfield, R. L. Mutations of the hexose-6-phosphate dehydrogenase gene rarely cause hyperandrogenemic polycystic ovary syndrome. *Steroids.* **1**(76), 135–139 (2011). 10.1016/j.steroids.2010.10.001 PMC3023921 21050867

[69] De Vita, S. *et al.* Insights into the ligand binding to bromodomain-containing protein 9 (BRD9): A guide to the selection of potential binders by computational methods. *Molecules* **26**(23), 7192 (2021). 34885774 10.3390/molecules26237192 PMC8659208

