# Supplementary material for: Piperine’s potential in treating polycystic ovarian syndrome explored through in-silico docking
Source: Sci Rep. 2024 Sep 18;14:21834. doi: 10.1038/s41598-024-72800-6 (PMC11411113; doi:10.1038/s41598-024-72800-6)
Supplement: Supplementary file 1 — Supplementary Material 1 [file 41598_2024_72800_MOESM1_ESM.docx]

**Piperine's Potential in Treating Polycystic Ovarian Syndrome Explored Through In-silico Docking**

**Supplementary figures**

**
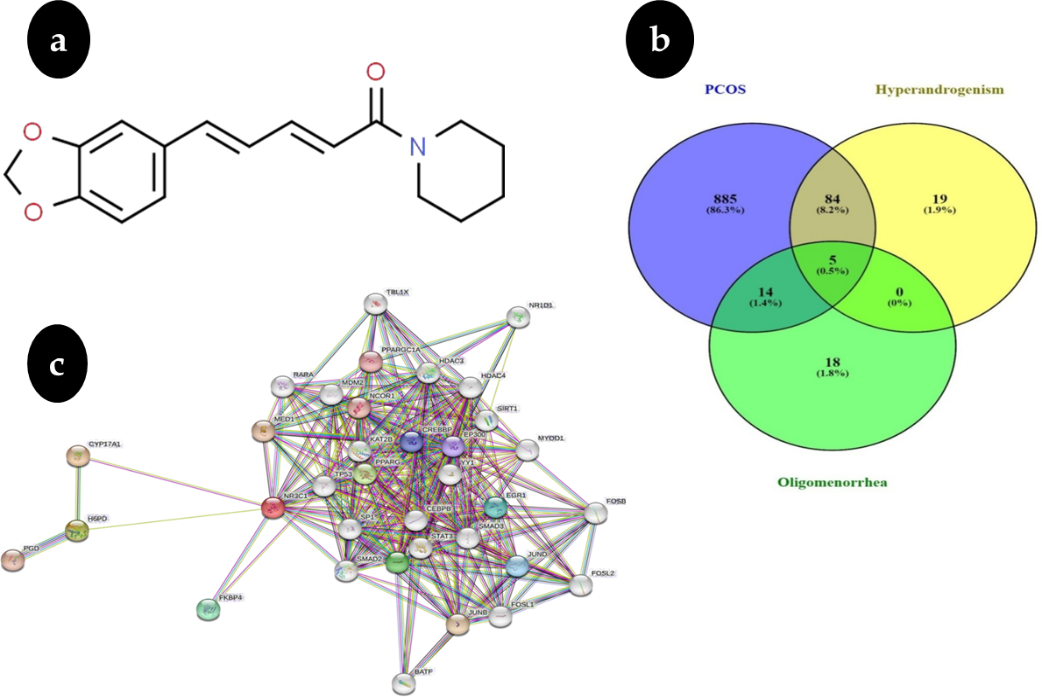
**

**Figure. S1: (a)** Piperine Chemical Structure **(b)** Targets in Poly Cystic Ovary Common Protein Syndrome **(c)** Protein-Protein (PPI) interaction network of common targets in PCOS


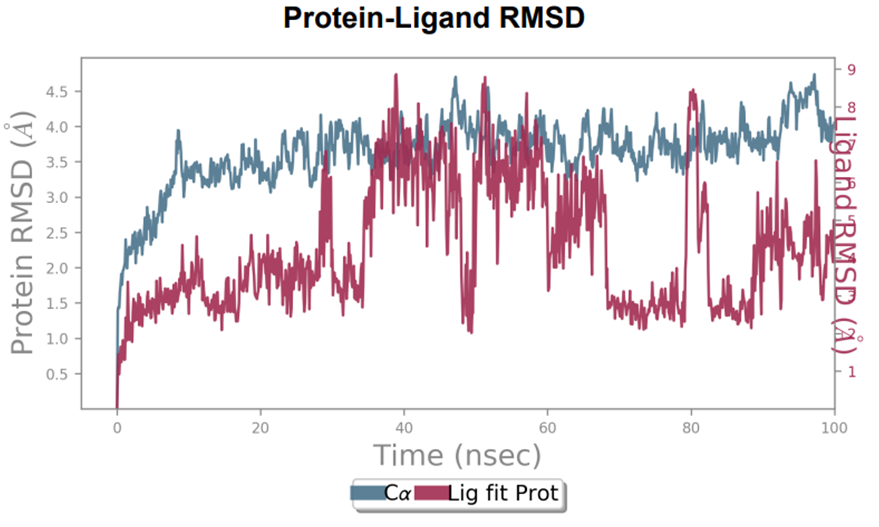


**Figure. S2:** The stability of the receptor-ligand complex is assessed by employing the root mean square deviation (RMSD) value.


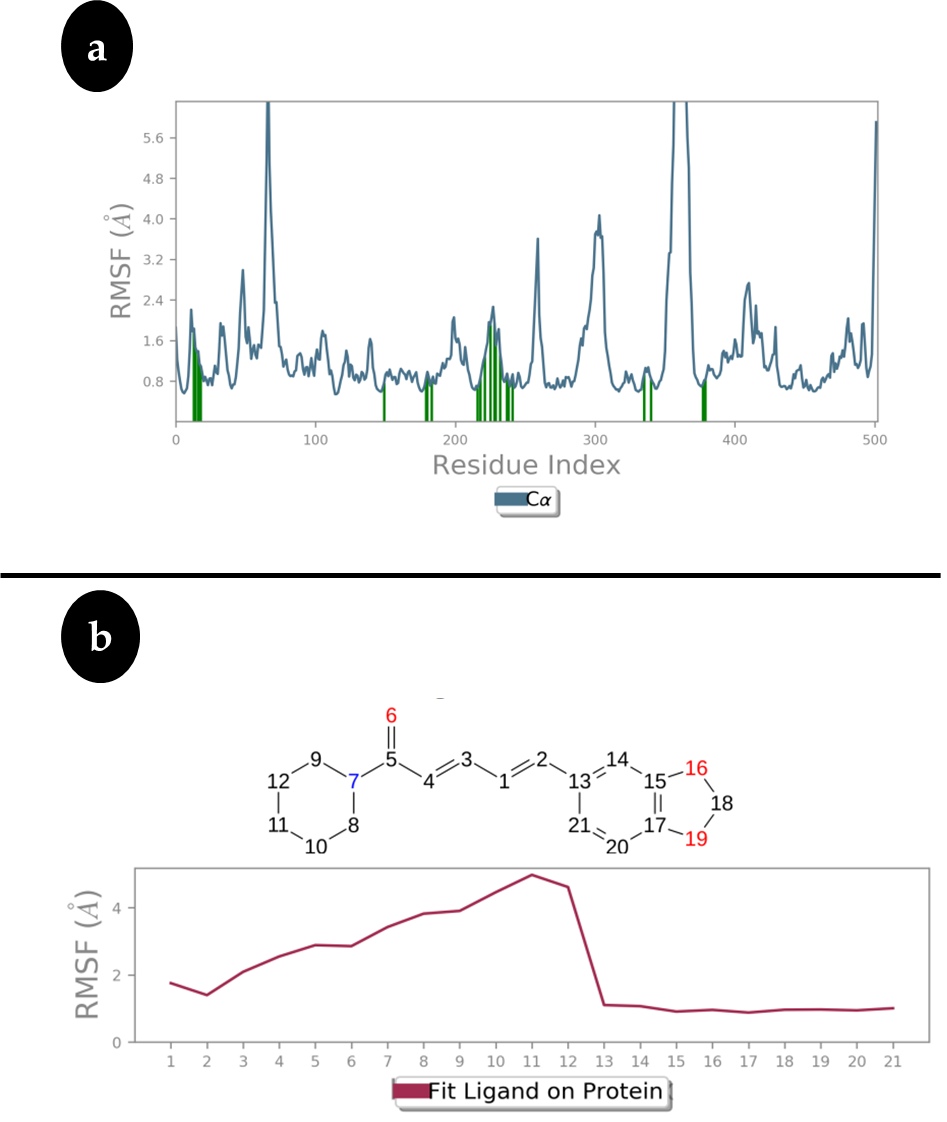


**Figure. S3: (a)** For ligand (Piperine), the average RMSF values of Hexose-6-phosphate dehydrogenase amino acid residues have been calculated using Desmond software **(b)** Fluctuation of the Ligand Root Means Square (L-RMSF)


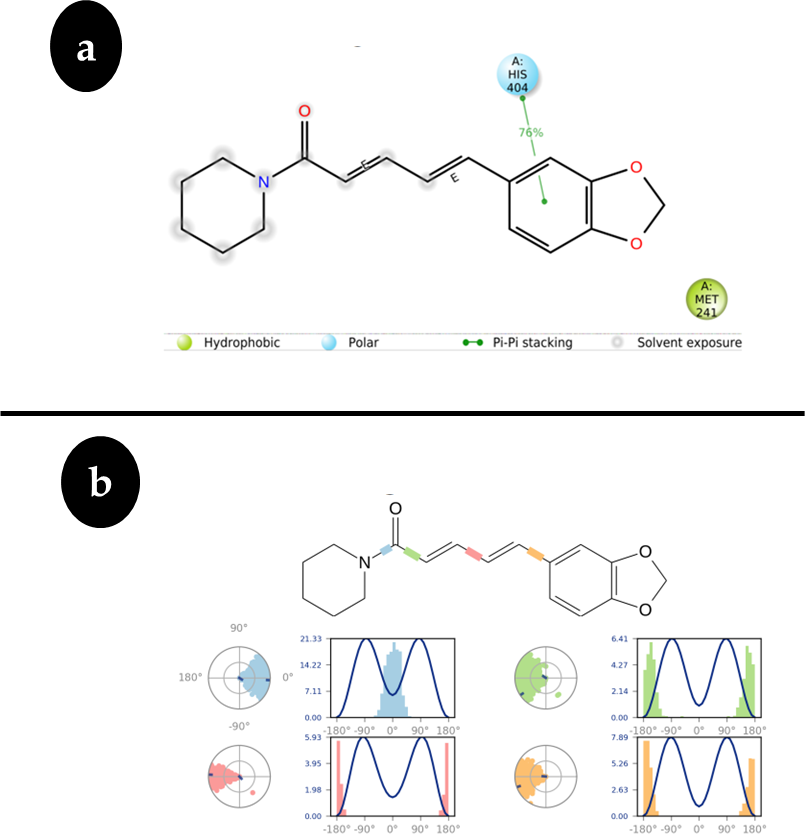


**Figure. S4: (a)** A thorough representation of the molecular reactions taking place inside the protein-ligand complex would be provided by a diagram showing the precise connections between the protein and ligand atoms. **(b)** The conformational development of potentially rotatable bonds is depicted by a ligand (Piperine) torsion map through the simulation study (100 ns).

**
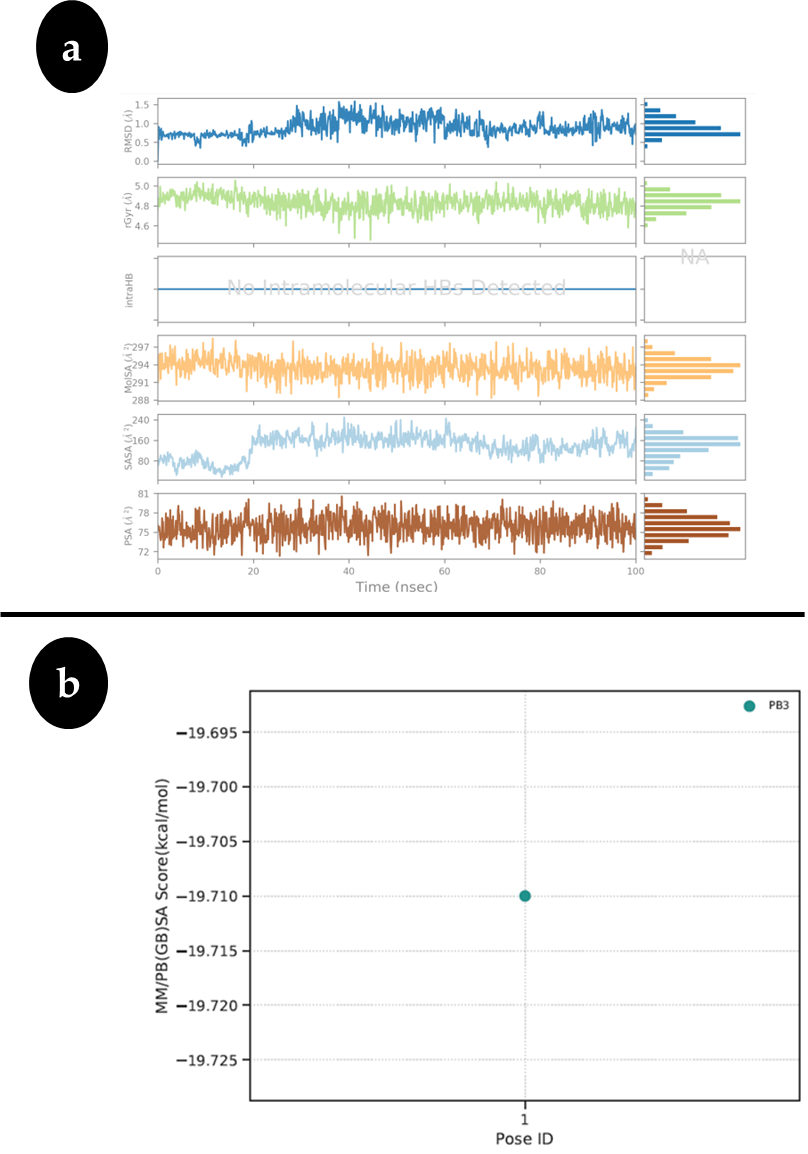
**

**Figure. S5: (a)** According to the RMSD ligand plot, the piperine has an RMSD value of 1.0. Å. **(b)** MM/PBSA calculation Plot of Hexose-6-phosphate dehydrogenase/Piperine docked complex.

**Supplementary Tables**

**Table. S1:** **ADMET Characteristics of Piperine**

| **Name** | **Expected Value** |
| --- | --- |
| **Absorptions** | |
| Water' dissolution | -3.464 mol/L |
| Caco2 permeation | 1.596 Papp in 10-6 cm/s |
| Gastro intestinal assimilation (human) | 94.444 % Absorbed |
| Epidermis Permeation | -3.131 Kp |
| Glycoprotein P substance | Yes |
| Glycoprotein P I inhibitory agent | Yes |
| Glycoprotein P II inhibitory agent | No |
| **Distributions** | |
| Volume of distribution | 0.158 L/kg |
| Portion of loosely bound (human) | 0.134 Fu |
| Blood-brain barrier (BBB) accessibility | -0.102 BB |
| Central nervous system (CNS) permeability | -1.879 PS |
| **Metabolisms** | |
| CYP2D6 substance | No |
| CYP3A4 substance | Yes |
| CYP1A2 inhibitory agent | No |
| CYP2C19 inhibitory agent | Yes |
| **Excretions** | |
| Total Clearance | 0.232 ml/min/kg |
| kidney OCT2 substance | Yes |
| **Toxicity** | |
| Max. permitted dosages (human) | -0.38 mg/kg/day |
| hERG I inhibitory agent | No |
| hERG II inhibitory agent | No |
| Acute oral Toxic effect rat (LD50) | 2.811 mol/kg |
| Chronic oral Toxic effect (LOAEL) | 1.51 mg/kg_bw/day |
| T.Pyriformis toxic effect | 1.879 μg/L |
| Minnow toxic effect | 1.732 Mm |

**Table. S2:** List of pathways of common protein targets ^31^.

| **Sr.No** | **Name** | **P-Value** | **Adjusted P-Value** |
| --- | --- | --- | --- |
| 1 | Prolactin signaling pathway | 0.0001199 | 0.006716 |
| 2 | Pentose phosphate pathway | 0.007478 | 0.05026 |
| 3 | Osteoclast differentiation | 0.0003951 | 0.01097 |
| 4 | Thyroid cancer | 0.009217 | 0.05026 |
| 5 | Non-alcoholic fatty liver disease | 0.0005877 | 0.01097 |
| 6 | Ovarian steroidogenesis | 0.01269 | 0.05026 |
| 7 | Lipid and atherosclerosis | 0.001126 | 0.01576 |
| 8 | Steroid hormone biosynthesis | 0.01516 | 0.05026 |
| 9 | Cortisol synthesis and secretion | 0.01615 | 0.05026 |
| 10 | Amphetamine addiction | 0.01713 | 0.05026 |
| 11 | PPAR signaling pathway | 0.01837 | 0.05026 |
| 12 | Pertussis | 0.01886 | 0.05026 |
| 13 | Leishmaniasis | 0.01910 | 0.05026 |
| 14 | Signaling pathway of B cell receptor | 0.02009 | 0.05026 |
| 15 | Colorectal cancer | 0.02132 | 0.05026 |
| 6 | The PD-1 checkpoint mechanism and PD-L1 expression in cancer | 0.02205 | 0.05026 |
| 17 | Differentiation of Th1 and Th2 cell | 0.02279 | 0.05026 |
| 18 | Rheumatoid arthritis | 0.02304 | 0.05026 |
| 19 | IL-17 signaling pathway | 0.02328 | 0.05026 |
| 20 | Circadian entrainment | 0.02402 | 0.05026 |
| 21 | Choline metabolism in cancer | 0.02426 | 0.05026 |
| 22 | Longevity regulating pathway | 0.02524 | 0.05026 |
| 23 | Signaling pathway of T cell receptor | 0.02573 | 0.05026 |
| 24 | Synthesis secretion and action Parathyroid hormone | 0.02622 | 0.05026 |
| 25 | Th17 cell differentiation | 0.02647 | 0.05026 |
| 26 | signaling pathway of TNF | 0.02769 | 0.05026 |
| 27 | Cholinergic synapse | 0.02794 | 0.05026 |
| 28 | Synthesis secretion and action Growth hormone | 0.02940 | 0.05026 |
